# Impact of local tumor-to-background uptake ratio on PET metabolic response assessment

**DOI:** 10.1093/rpd/ncaf145

**Published:** 2026-03-13

**Authors:** Esmaeil Mehrara, Mariam Mohamed, Martijn Van-Essen, Jesus Lopez Urdaneta

**Affiliations:** Department of Medical Physics and Biomedical Engineering, Sahlgrenska University Hospital, Blå stråket 5, målpunk G, plan 1, SE-413 45, Gothenburg, Sweden; Department of Medical Radiation Sciences, Gothenburg University, Radiofysikhuset, Gula stråket 2B, Sahlgrenska universitetssjukhuset, SE-413 45, Gothenburg, Sweden; Department of Medical Radiation Sciences, Gothenburg University, Radiofysikhuset, Gula stråket 2B, Sahlgrenska universitetssjukhuset, SE-413 45, Gothenburg, Sweden; Department of Clinical Physiology, Sahlgrenska University Hospital, Blå stråket 5, målpunk G, plan 1A, SE-413 45, Gothenburg, Sweden; Department of Clinical Physiology, Sahlgrenska University Hospital, Blå stråket 5, målpunk G, plan 1A, SE-413 45, Gothenburg, Sweden

## Abstract

Positron Emission Tomography Response Criteria in Solid Tumors (PERCIST) quantifies changes in radiotracer uptake to assess therapeutic response in cancer. However, the accuracy of these quantifications depends on imaging parameters, tumor size, and the local tumor-to-background uptake ratio (local-TBR). In this study, ‘background’ refers to the surroundings of the lesion rather than a standardized reference tissue. A NEMA Image Quality phantom was filled with ^18^F-FDG at varying sphere-to-background ratios to simulate clinical scenarios corresponding to PERCIST-defined thresholds for partial metabolic response (−30%) and progressive metabolic disease (+30%). Positron emission tomography (PET)/computed tomography imaging revealed that measured uptake changes systematically underestimated the true ±30% differences, particularly in smaller spheres. These findings indicate a potential source of systematic error in PET-based tumor response assessment, which may influence clinical interpretation. Further studies are recommended to investigate the effects of varying imaging parameters, tumor types, and clinical settings to improve the robustness of PERCIST-based evaluations.

## Introduction

Positron emission tomography (PET) is a nuclear medicine imaging technique used to visualize and quantify biological processes. It involves administering a positron-emitting radiotracer and imaging its distribution within the patient’s body. Radiotracers are designed to target specific biological pathways. For example, ^18^F-FDG accumulates in metabolically active tissues, making it ideal for oncologic imaging. Other examples include ^68^Ga-DOTATOC/TATE for neuroendocrine tumors and ^68^Ga-PSMA-11 for prostate cancer [1].

Hybrid imaging modalities such as PET/CT (computed tomography) and PET/magnetic resonance imaging (MRI) have become essential in oncology. PET provides functional information by visualizing metabolic activity, while CT contributes to high-resolution anatomical detail. Their combination enables precise localization of radiotracer uptake and improves diagnostic accuracy. A major advantage of PET/CT is attenuation correction (AC), which compensates for signal loss due to tissue absorption and scattering. CT-derived attenuation maps enhance the quantitative accuracy of radiotracer distribution, improving image fidelity and reproducibility [2]. PET/CT is widely used to detect tumors and metastases, differentiate malignant from benign lesions, determine disease stage, and monitor treatment response. Its ability to integrate metabolic and structural data makes it a powerful tool for personalized cancer care [3].

Accurate quantification of radiotracer uptake is essential in oncologic PET for diagnosis, staging, and treatment monitoring. PET visualizes metabolic activity by detecting radiotracers that accumulate in tissues based on their biochemical properties. Among the available metrics, standardized uptake values (SUV) are the most widely used for quantifying tumor burden and assessing treatment response. SUV is a semiquantitative metric that normalizes radiotracer activity concentration within the volume of interest (VOI) to the injected activity and patient body weight (or lean body mass, in the case of SUL). SUVs are dimensionless and assume tissue density equivalent to water (1 g/ml), providing a standardized method for comparing uptake across patients and time points [4].

Common SUV metrics include:

SUV_max_: the highest voxel value within the VOI; widely used due to its simplicity but highly sensitive to noise [5].

SUV_mean_: the average uptake within the VOI; more stable but dependent on VOI delineation [6].

SUV_peak_: the average uptake within a 1 cm^3^ spherical volume centered on the hottest region; less sensitive to noise and more reproducible than SUV_max_ but may underestimate uptake in small lesions [7].

Each metric has strengths and limitations depending on lesion size, image noise, and clinical context.

Reliable SUV measurements are crucial for clinical decision-making, enabling consistent evaluation of disease progression or response. However, SUV accuracy is influenced by numerous factors, including scanner resolution, calibration, reconstruction algorithms, and patient-specific variables such as body composition and injected activity. To mitigate these factors, standardized imaging protocols and quality assurance procedures have been developed, supported by organizations such as SNMMI and EANM [8].

The PERCIST provides a standardized framework for evaluating metabolic changes using PET imaging. It relies on quantitative SUV measurements to assess treatment response over time [9]. PERCIST classifies response into four categories:

- Complete response (CR): complete resolution of metabolic activity in the lesion with no new lesions.
- Partial response (PR): ≥30% decrease in SUV and ≥ 0.8 unit decrease in SUL_peak_ (or 0.9 for SUV_peak_), with no new lesions.
- Stable disease (SD): no significant change in SUV.
- Progressive disease (PD): ≥30% increase in SUV and ≥ 0.8 unit increase in SUL_peak_ (or 0.9 for SUV_peak_), or appearance of new lesions [10].

These thresholds are designed to reflect true biological changes while minimizing misclassification due to measurement variability.

Phantom studies have shown that SUV measurements can vary significantly with tumor-to-background ratios, potentially leading to misinterpretation of response. Changes in this ratio, whether due to technical or biological factors, may cause observed SUV changes to deviate from actual biological effects, impacting PERCIST-based evaluation [11, 12]. In most such studies, the background SUV is measured in spherical volumes of interest placed in physiological liver tissue.

This study investigates how post-therapy changes in local tumor-to-background uptake ratios (local-TBR) affect SUV measurements and their interpretation under PERCIST. It is important to note that in this study, ‘background’ refers to the surroundings of the lesion or sphere, rather than a reference tissue such as the liver, as used in previous studies. Using a NEMA IQ phantom, Data Spectrum Corporation, USA, we simulate clinical scenarios with varying sphere-to-background ratios (SBRs), modeling clinical Local-TBR variations, to assess their impact on quantification accuracy and response classification.

The aim of this study was to evaluate how changes in Local-TBR between baseline and post-therapy conditions influence the accuracy of SUV-based treatment response assessments in PET imaging. Specifically, we investigated whether such variations could lead to misclassification of metabolic response according to PERCIST criteria.

## Materials and Methods

### Phantom preparation

A NEMA Image Quality (IQ) phantom was used to simulate tumor lesions of varying sizes and uptake levels. The phantom contains six fillable spheres with diameters of 10, 13, 17, 22, 28, and 37 mm, embedded in a 9.8-liter water-filled background. A low-density insert mimicking lung tissue was positioned centrally. To model clinical response scenarios as defined by PERCIST (±30% SUV change), the phantom was filled with seven different sphere-to-background activity concentration ratios (SBRs): 2.00, 2.60, 3.71, 4.83, 6.90, 8.97, and 12.81.

The radiotracer used was ^18^F-FDG. Imaging was performed when the background activity reached approximately 2 kBq/ml (Table 1).

**Table 1:** SBR in a phantom to simulate ±30% post-therapy changes in local tumor-to-background ratio (local-TBR) in a clinical setting. Note that the background in this study refers to the filled body of the phantom which surrounds the spheres.

SBR	2.00:1	2.60:1	3.71:1	4.83:1	6.90:1	8.97:1	12.81:1
Ratio-relationship	Baseline SBR	2.00 + 30% 3.71-30%	Baseline SBR	3.71 + 30% 6.90-30%	Baseline SBR	6.90 + 30% 12.81-30%	Baseline SBR

### Positron emission tomography/computed tomography imaging

Imaging was performed using a GE Discovery MI PET/CT system, employing standard clinical protocols for ^18^F-FDG. The phantom was positioned with the spheres facing the gantry and centered using the system’s laser alignment tools. A CT scan was first acquired for attenuation correction, followed by a static PET acquisition with a scan duration of 20 minutes per dataset.

The following PET reconstruction parameters were applied: time-of-flight (TOF), point-spread function (PSF), and ordered subset expectation maximization (OSEM) with 4 iterations, 16 subsets, and a 6 mm Gaussian post-reconstruction filter. The GE Bayesian penalized-likelihood reconstruction algorithm (Q.Clear) was not used in this study to ensure harmonization with current standard recommendations and maintain consistency with our previous quantitative phantom work.

### Image analysis

Image analysis was performed using Syngo.via (Siemens Healthineers). The following SUV metrics were extracted:

- SUV_max_: maximum voxel value within each sphere
- SUV_peak_: average activity concentration within a 1 cm^3^ volume centered on the hottest region
- SUV_mean_: calculated using a 50% isocontour VOI.

Background SUV was determined by averaging the values from six VOIs placed in the homogeneous background region of the phantom. The same global background value was used for all spheres. SUV ratios (SUVR) were then computed by dividing each sphere’s SUV by this background SUV. These SUVRs were then compared across phantom conditions simulating ±30% changes in SBR.

## Results 

**Figure 1:**
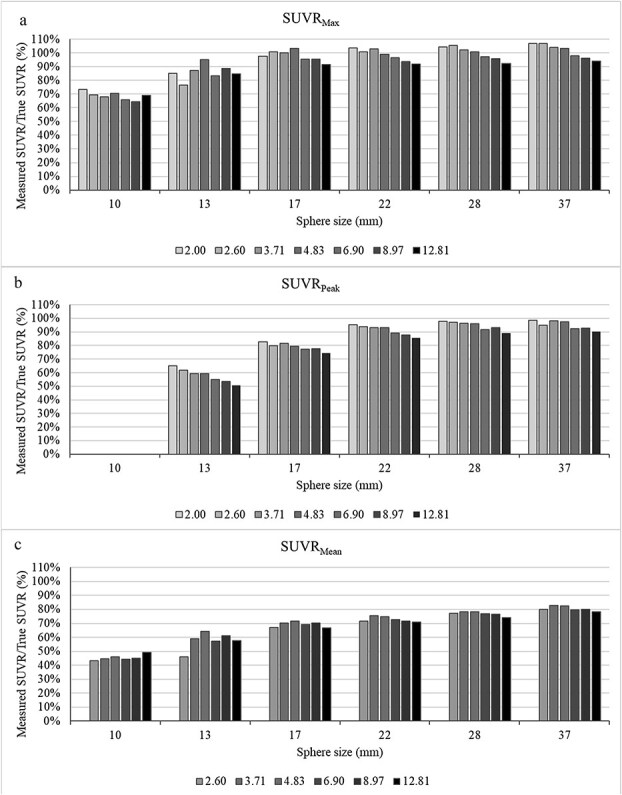
Measured SUVR_max_ (a), SUVR_peak_ (b), and SUVR_mean_ (c) for true SUVR levels of 2.00, 2.60, 3.71, 4.83, 6.90, and 12.81 and sphere diameters of 10, 13, 17, 22, 28, and 37 mm. A general downward trend in measured SUVR is observed with increasing true SUVR, specially for SUVR_peak_. Note that data from 10 mm sphere is not included for SUVR_peak_, because the size is smaller than the required size by SUVR_peak_ definition. The SUVR_mean_ equal to 2 is also excluded, because the 50% thresholds are equal to the background.

Measured SUVR_max_, SUVR_peak_, and SUVR_mean_ for true SUVR levels of 2.00, 2.60, 3.71, 4.83, 6.90, and 12.81 across sphere diameters of 10, 13, 17, 22, 28, and 37 mm are presented in Fig. 1. A general downward trend in measured SUVR values is observed with increasing true SUVR, particularly for SUVR_peak_. Among the three metrics, SUVR_max_ consistently produced the highest values, followed by SUVR_peak_ and SUVR_mean_. For the smallest spheres, SUVR_mean_ occasionally exceeded SUVR_peak_ due to partial volume effects and background inclusion within the VOI.

It should be noted that the 10 mm sphere data were excluded from SUVR_peak_ analysis, as its size is below the minimum volume required by the metric’s definition (1 cm^3^). Similarly, SUVR_mean_ data for SUVR = 2.00 were excluded because the 50% isocontour threshold corresponds to the background level, causing the VOI to extend into the background region.

**Figure 2:**
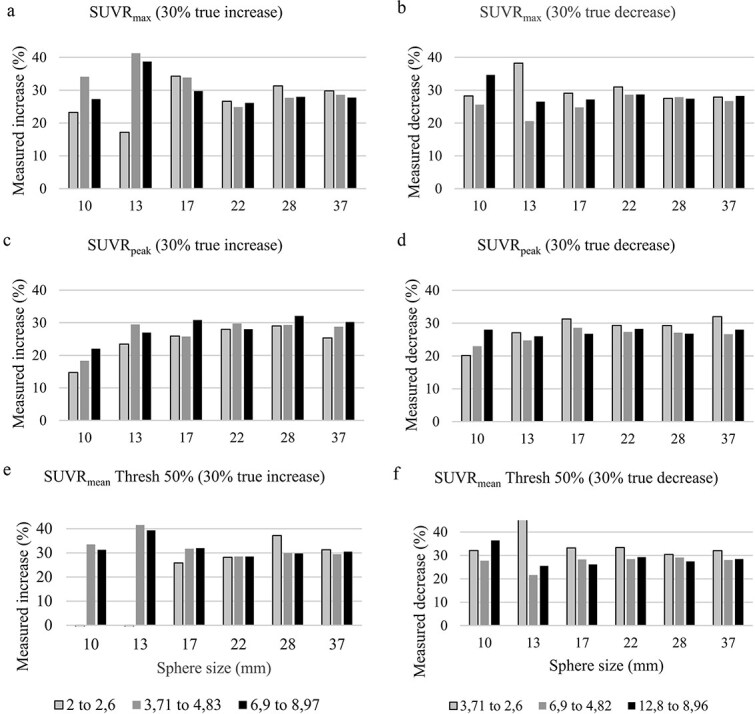
Measured SUVR_max_ (a), SUVR_Peak_ (c), and SUVR_mean_ (e) increases for true SUVR increase of 30% for sphere diameters of 10, 13, 17, 22, 28, and 37 mm. Measured SUVR_max_ (b), SUVR_peak_ (d), and SUVR_mean_ (f) decreases for true SUVR decrease of 30% for sphere diameters of 10, 13, 17, 22, 28, and 37 mm. Note that data from 10 mm sphere is not included for SUVR_peak_, because the size is smaller than the required size by SUVR_peak_ definition. The SUVR_Mean_ equal to 2 is also excluded, because the 50% thresholds are equal to the background.

Measured SUVR_max_, SUVR_peak_, and SUVR_mean_ values corresponding to a true ±30% change in SUVR for each sphere size are shown in Fig. 2. In general, the measured changes underestimated the true ±30% variation, especially at lower SUVR levels. Among the three metrics, SUVR_peak_ demonstrated the most consistent performance across different sphere sizes and uptake levels.

**Figure 3:**
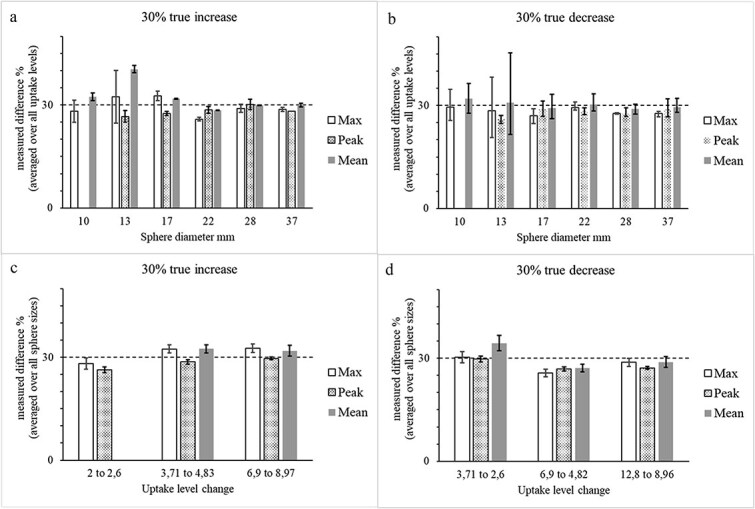
Measured SUVR_max_, SUVR_peak_, and SUVR_mean_ increases/decrease (a/b) averaged over all SUVR levels for each sphere size for true increase/decrease of 30%. Measured SUVR_max_, SUVR_peak_, and SUVR_mean_ increases/decrease (d/d) averaged over all sphere sizes for each uptake/SUVR level for true increase/decrease of 30%. Note that data from 10 mm sphere is not included for SUVR_peak_, because the size is smaller than the required size by SUVR_peak_ definition. The SUVR_mean_ equal to 2 is also excluded, because the 50% thresholds are equal to the background.

Figure 3 presents the average measured increases and decreases in SUVR_max_, SUVR_peak_, and SUVR_mean_, grouped (a) by sphere size across all SUVR levels and (b) by SUVR level across all sphere sizes. These trends were consistent with those observed in Fig. 2, showing systematic underestimation of true uptake changes, particularly in smaller spheres and lower SBR scenarios.

These findings indicate that when post-therapy changes in tumor uptake are used to assess therapeutic efficacy in FDG PET, especially under PERCIST criteria, partial volume effects and low Local-TBR may cause underestimation of both increases and decreases in uptake. Consequently, patients with progressive metabolic disease (PMD) or Partial Metabolic Response (PMR) may be misclassified as having SD due to insufficiently detected change in measured uptake.

## Discussion

This study evaluated the impact of varying local-TBR on SUV-based quantification in PET. The clinical scenarios simulated with SBRs in a NEMA phantom filled with a homogeneous background and spheres with different sizes, which simulated tumors in a surrounding tissue. The results demonstrated that the measured standardized uptake value ratios (SUVR) in relation to true SUVR increased with sphere size approaching the true values for larger spheres, as was shown before [13]. On the other hand, measured-SUVR/true-SUVR in each sphere decreased by increasing true SBR. This indicates that the true value of each unit of measured SUVR changes when SBR changes. Change in Local-TBR in patients, simulated by SBR in this study, can occur through change in tumor uptake (SUV) due to biological response (progression or regression), and/or change in background uptake in tissue that surrounds the tumor due to various physiological or technical factors (e.g. inflammation, therapy effects, tracer biodistribution).

It is important to emphasize that in this study, ‘background’ refers to the immediate surroundings of the lesion (sphere) rather than a reference tissue such as the liver, as used in PERCIST. Our aim was to isolate how local background uptake affects tumor SUV quantification. This information is complementary to clinical PERCIST workflows, where the measured tumor SUV is ultimately interpreted in relation to a stable reference region such as the liver.

When PERCIST is used for tumor response assessment, post-therapy tumor uptake (SUV or SUL) is compared to baseline, with the implicit assumption that the measured change accurately reflects the true biological change in metabolic activity. Our results demonstrate that this assumption can be violated when TBR ratios change, where background refers to the actual surroundings of tumor. The measured change in uptake is systematically underestimated, particularly for small lesions and low SBRs. For example, a true ±30% biological change—which defines partial metabolic response or progression in PERCIST—may be measured as <30% and therefore incorrectly classified as stable metabolic disease. Such misclassification risk extends beyond PERCIST to any clinical evaluation where lesion uptake is interpreted in relation to reference regions, such as anatomical response, pathology, or survival endpoints.

A significant factor affecting SUL/SUV measurements is the partial volume effect (PVE) which mainly affects smaller lesions especially those smaller than two times of the systems spatial resolution [14]. PVE leads to SUVR being underestimated for small spheres and increases with sphere size. This phenomenon is called spill-in and spill-out effects, which have a significant impact on the quantification accuracy of PET imaging [15]. Since the resolution of the PET scanner is limited the signal from small spheres will spread to surrounding voxels, resulting in the spill-out effect. This effect means that the measured radioactivity in the sphere is lower than the true SUVR values [14, 15].

Different types of SUV metrics, including SUV_max_, SUV_peak_, and SUV_mean_, are used to describe different aspects of the radiotracer uptake. SUVR_max_ gave the highest values in all spheres as well as for all SBR compared to other SUVR metrics. SUV_max_ is often used in the clinic because it is easy to determine and gives an indication of the most metabolically active part of a lesion. A limitation of SUV_max_ is its susceptibility to image noise and its reliance on a single voxel, which prevents it from reflecting the lesion’s overall metabolic activity. Analysis of the additional SUV metrics showed that for the four largest spheres, SUVR_peak_ values were higher than SUVR_mean_, whereas for the two smallest spheres, SUVR_mean_ exceeded SUVR_peak_. With a volume of 0.5 cm^3^, the smallest sphere falls below the 1 cm^3^ volume used to define SUVR_peak_, causing the peak metric to incorporate background activity outside the sphere, whereas SUVR_mean_ better reflects the true sphere volume. However, SUV_means_ are not considered useful at low SBRs such as 2:1, because at these low SBRs, the SUV_mean_ threshold of 50% of SUV_max_ means that the mean includes almost all background, since 50% of SUV_max_ corresponds to the background level. Even at SBR 2.6:1, the SUV average was found not to be good for small spheres with diameters of 10, 13 and 17 mm.

In sufficiently large spheres, SUVR_peak_ becomes higher than SUVR_mean_ because it focuses on the most active region within a volume of 1 cm^3^, while SUVR-_mean_ includes both high- and low-activity regions in the VOI. This means that SUVR_peak_ better reflects the highest activity concentration in larger lesions, more heterogeneous lesions, while SUVR_mean_ gives a more comprehensive picture of the total metabolic activity within the entire VOI but may underestimate the most active part in lesions. In addition, SUV_peak_ is less affected by noise level than SUV_max_ because this VOI comprises several pixels, making it less sensitive to random variations in the image data and a more robust and reliable measure [16].

SUV_mean_ represents the average uptake across the entire VOI and is the least sensitive to noise among the three metrics. Because it incorporates many voxels, random variations in individual pixels are minimized, making SUV_mean_ more robust and reliable [17]. However, SUV-mean was determined by drawing the VOI based on SUV_max_. The disadvantage of this approach is that the resulting region affected by the noise in SUV_max_ [18]. A major weakness of using SUV_max_-based VOI to determine SUV_mean_ is that the selected volume can become larger than the lesion itself and thus include background tissue [19], especially at low SBR values. This can lead to underestimation of measured values, especially for small lesions/spheres where noise effects are high. Consequently, the percent change of SUVR_mean_ for small spheres with low SBR showed negative values, due to this source of error. The 2:1 SUVR has been excluded from results.

Although this study focused primarily on ^18^F-FDG, the findings are highly relevant for other PET tracers. For instance, ^68^Ga-based radiotracers (e.g. DOTATOC, PSMA) exhibit longer positron ranges and lower positron yields, which further exacerbate partial volume effects and reduce spatial resolution [20–22]. These physical differences make ^68^Ga-derived SUVR measurements more sensitive to background changes and lesion size, compounding the issues observed with ^18^F-FDG. Therefore, in clinical situations using ^68^Ga-labeled tracers—especially for small lesions or heterogeneous uptake patterns—the interpretation of SUVR-based metrics should be approached with caution.

It should be noted that some PERCIST criteria were not applied in this study, including the requirement that baseline SUV must be ≥1.5 × liver SUV_mean_ + 2 SD, and that metabolic response requires both a ≥ 30% change and an absolute SUL change of ≥0.8 units. These criteria were omitted deliberately, as our primary objective was to isolate and characterize quantification bias caused by varying local-TBR uptake ratios, where background refers to the tissue that is surrounding the tumor. While this controlled phantom approach strengthens internal validity, it simplifies some aspects of clinical tumor response assessment. Therefore, care should be taken when extrapolating the magnitude of observed effects directly to patient studies, where physiological variability in liver or blood-pool uptake will also contribute to PERCIST decision-making. The specific impact across tumor types and clinical scenarios warrants further investigation.

## Conclusion

This study demonstrates that variations in local-TBR uptake ratios can bias SUV-based quantification and metabolic response assessment in PET, particularly when applying PERCIST criteria. Further work is needed to develop standardized approaches that account for these effects and strengthen the robustness of quantitative PET and PERCIST-based evaluations.
